# Antibiotic Prescribing among Pediatric Inpatients with Potential Infections in Two Private Sector Hospitals in Central India

**DOI:** 10.1371/journal.pone.0142317

**Published:** 2015-11-05

**Authors:** Megha Sharma, Anna Damlin, Ashish Pathak, Cecilia Stålsby Lundborg

**Affiliations:** 1 Department of Pharmacology, Ruxmaniben Deepchand Gardi Medical College, Ujjain, India; 2 Department of Public Health Sciences, Global Health—Health Systems and Policy (HSP): Medicines, focusing antibiotics, Karolinska Institutet, Tomtebodavägen 18A, 17177 Stockholm, Sweden; 3 Department of Pediatrics, Ruxmaniben Deepchand Gardi Medical College, Ujjain, India; 4 Department of Women and Children’s Health, International Maternal and Child Health Unit, Uppsala University, SE 751 85 Uppsala, Sweden; The Hospital for Sick Children and The University of Toronto, CANADA

## Abstract

**Introduction:**

Infectious diseases are one of the major causes of child mortality in India. Pediatric patients are commonly prescribed antibiotics for non-bacterial infections. Monitoring of local antibiotic prescribing with respect to the diagnosis is necessary to improve the prescribing practices. The aim of the study was to describe antibiotic prescribing for potential infections among patients admitted in pediatric departments in two private sector hospitals; one teaching (TH) and one non-teaching (NTH) in Central India.

**Methods:**

Data from all patients admitted at the pediatric departments of both study hospitals was collected manually, for 3 years (2008–2011) using a customized form. Data from inpatients aged 0–18 years, diagnosed with; acute gastroenteritis (AGE), respiratory tract infections, enteric fever, viral fever or unspecified fever were focused for analysis. Antibiotic prescriptions were analysed using the WHO Anatomical Therapeutic Chemical (ATC) classification system and defined daily doses (DDDs). Adherence to the Indian Academy of Pediatrics list of essential medicines (IAP-LEM) was investigated. P-values <0.05 were considered significant.

**Results:**

Oftotal6, 825 inpatients admitted at two pediatric departments, 510 patients from the TH and 2,479from the NTH were selected based on the assigned potential infectious diagnoses. Of these, 224 patients (44%) at the TH and 2,088 (84%) at the NTH were prescribed at least one antibiotic during hospital stay (odds ratio-0.69, 95%confidence interval-0.52 to 0.93; p<0.001). Patients with AGE, viral- and enteric fever were frequently prescribed antibiotics at both hospitals, yet higher proportion were prescribed antibiotics at the NTH compared to the TH. Broad-spectrum antibiotics were the most commonly prescribed antibiotic class in both hospitals, namely third generation cephalosporins, J01DD (69%) at the TH, and new fixed dose combinations of antibiotics J01R (FDCs, 42%) at the NTH. At the TH, 37% of the antibiotic prescriptions were comprised of antibiotics listed in the IAP-LEM, compared to 24% at the NTH (p<0.05).

**Conclusions:**

Broad-spectrum antibiotics were prescribed frequently in both hospitals also for the un-indicated conditions such as viral fever and enteric fever. At the NTH, new FDCs were more frequently prescribed and adherence to the IAP-LEM was substantially lower at the NTH compared to the TH. The results demonstrate need to develop diagnosis-specific prescribing guidelines to facilitate rational use of antibiotics and implement antibiotic stewardship program.

## Introduction

Antibiotic resistance, which is a threat to public health, is rapidly increasing globally [1].

Use of antibiotics, including irrational and unnecessary antibiotic treatment, contributes to the development of antibiotic resistance [1,2].

Infectious diseases are common among pediatric patients in India and contribute to the total mortality rate, which is the highest in the world [3]. Pneumonia and diarrhoeal diseases account for 50% of total 1.34 million deaths among Indian children between 1 month to 5 years of age [3]. Appropriate use of antibiotics is vital in reducing the mortality caused by bacterial infections [1]. However, antibiotics are often prescribed inappropriately for un-indicated conditions. Studies from India have shown antibiotics as one of the most commonly prescribed drug classes in pediatric patients [4–5]. Despite the fact that high proportions of acute respiratory tract infections and diarrhoea in pediatric patients are caused by viruses and should not be treated with antibiotics; patients with diarrhoea were prescribed antibiotics in India [5–7].

The World Health Organisation (WHO) advises implementing national lists of essential medicines with assured quality for rational use of medicines [1]. In order to avoid unnecessary antibiotic prescribing and development of antibiotic resistance, pediatric healthcare facilities in India are advised to follow the Indian Academy of Pediatrics list of essential medicines (IAP-LEM) [8]. The IAP-LEM includes 16 different antibiotics with recommended dosage and route of administration for children in India but is not a diagnosis specific guideline.

Analyses of antibiotic prescription practices provide the basis for development of diagnosis specific antibiotic prescribing guidelines, which contribute to appropriate use of these life- saving medicines [9,10]. According to the WHO, it is important to document antibiotic prescribing practices among in-patients and compare it with other hospitals to identify areas for intervention for rationalizing prescribing [11,12]. Some studies from India have shown overall higher antibiotic prescribing rates at private sector health care facilities compared to public sector facilities [13–16]. The private sector hospitals are major health service providers in India, yet, most Indian studies of antibiotic prescribing practices among pediatric patients have been conducted among out-patients and at public health care facilities [4–6, 15,16]. Absence of studies that links antibiotic prescribing with the indication or focus of infection at pediatric inpatient departments is one of the main barriers to estimate the extent of over- or under- prescribing of antibiotics. Thus, for the present study, we have compared antibiotic prescribing patterns for the patients admitted with a diagnosis of potential infection, in pediatric departments at two private sector hospitals, and to identify the areas for improvement in antibiotic prescribing practices.

## Methods

### Study setting

An observational study was conducted in the pediatric departments at two tertiary care private sector hospitals, one teaching hospital (TH) and one non-teaching hospital (NTH), in Ujjain district, India. The TH is located in a village outside Ujjain city and has 570 beds of which 60 are pediatric beds and a 6-bed, level II neonatal intensive care unit (NICU) at the time of study. The TH is attached to Ruxmaniben Deepchand Gardi Medical College which provides both undergraduate and post graduate medical education. The NTH is located in the city centre and has 350 beds including 30 were pediatric beds and15-bed, level II NICU. The consultants at the NTH receive payments per patient admitted by them, while in the TH consultants have a fixed monthly salary. Although both hospitals are run by the same trust, the patients at the TH are provided with medical services and medicines free of charge, while at the NTH the patients have to pay for the services (subsidized for all patients) and purchase the prescribed medicines. Visits of representatives of pharmaceutical companies were restricted at the TH while there was no such restriction at the NTH.

### Study participants

Antibiotic prescribing data was collected prospectively for all patients admitted at pediatric departments at the TH or at the NTH between April 1^st^, 2008 and March 31^st^, 2011. The 'pediatric group' is defined as persons below 18 years of age and the available lists and recommendations for rational prescribing of antibiotics are not specified according to ages and thus are applicable for all pediatric patients. Therefore, the patients below 18 years of age that stayed for at least one night were included in the analysis.

### Data collection, management and analysis

A data collection form was attached to the patient record file of each patient admitted to the pediatric wards in the two hospitals [15,16]. The form required information about the patient’s age, sex, diagnosis, duration of hospital stay; if antibiotics were prescribed or not during the hospital stay, duration of antibiotic treatment, type of prescribed antibiotic, dose, frequency, and route of administration. The forms were filled in by nursesemployed by the hospital and were a part of routine patient care practices. The nurses were trained for data collection. The diagnoses were recorded at the time of discharge as stated by the consultant in-charge in the patient record file. The diagnoses made by the consultant in-charge were considered as final diagnosis and were not verified externally.

The antibiotic prescribing data included in the analysis was from patients who had one of these five most commonly registered potential infectious diagnoses acute gastroenteritis (including bloody diarrhoea); respiratory tract infections (RTIs including both upper and lower respiratory tract infections, pneumonia and bronchitis); enteric fever; viral fever; or unspecified fever. Various causes of fever were included in the analysis because fever is an important cause of morbidity of children across pediatric age groups and also a pointer to a potential infectious etiology. The prescribed antibiotics were classified according to the WHO Collaborating Centre for Drug Statistics Methodology (WHOCC), Anatomical Therapeutic Chemical (ATC) classification with defined daily dose (DDD) [17,18]. All DDDs were calculated as DDD per 1000 patients per diagnosis group.

ATC codes were followed up to the 7^th^ level. For those drug combinations where no ATC code was available from the WHOCC list, advice from WHOCC was followed and the combinations were coded with individual codes according to the WHO guidelines. The drug utilisation (DU) 90% methodology was used to present the antibiotics accounting for 90% of all antibiotics prescribed [19].

The manually collected antibiotic prescribing data was entered in EPI Info 3.1 and analysed using STATA software version 13.0 (Stata Corp., College Station, Texas, USA) and SPSS Statistics version 22.0 (SPSS Inc, Chicago, IL, USA) for further analysis. Crude odds ratios (OR) with their 95% confidence intervals (CI), were calculated from two by two tables using SPSS software. Frequency and percentage were calculated for categorical variables. Sum, median, mean, range and standard deviations were calculated for continuous (numerical) variables. The independent samples t-test was used for comparison of continuous (numerical) variables, after checking for a normal distribution. The Chi-square test was used for comparisons of categorical values. P-values <0.05 were considered significant.

A unique code was generated for each patient record to anonymize and analysis was conducted at the group level without identifying the patients individually. The study was approved by the ethics committee of Ruxmaniben Deepchand Gardi Medical College, Ujjain, India (approval number: 41/2007).

## Results

During the study period overall 6825 patients were admitted in two study hospitals; 1,977 patients were admitted to the pediatric ward at the TH and 4,848 to the NTH (Table 1). Out of these, 510 patients (26%) in the TH and 2,479 (51%) in the NTH were diagnosed with one of the five most common infectious diagnoses and were included for further analysis. In the TH 44% (244/510) and in the NTH 84% (2,088/2,479) of children with five selected diagnoses were prescribed at least one antibiotic (OR 0.69, 95% CI 0.52 to 0.93; p<0.001). Table 2 shows the distribution of patients and antibiotic prescribing for the selected diagnoses.

**Table 1:** Antibiotic prescribing, duration of hospital stay and antibiotic treatment duration of all patients admitted at the pediatric departments in a teaching and a non-teaching hospital in Central India.

	TH			NTH			P-value^@^
	Male	Female	Total	Male	Female	Total	
**All patients admitted in pediatric department; N**	1135	842	1977	3430	1418	4848	-
**Inpatients prescribed AB; n (%)**	383 (34)	296 (35)	679 (34)	2836 (83)	1127 (79)	3963 (82)	<0.001
**Duration of hospital stay; mean days (SD)**	6.8 (6)	6.6 (5)	6.7 (5.7)	4.6 (3.07)	4.4 (2.8)	4.5 (3)	<0.001
**Duration of AB treatment; mean days (SD)**	7.1 (4.9)	6.8 (3.9)	6.6 (4.6)	4.4 (2.7)	4.4 (2.6)	4.4 (2.7)	<0.001

Abbreviations: TH = teaching hospital; NTH = non-teaching hospital; N = Overall number of inpatients, n = number of inpatients; AB = antibiotics; SD = standard deviation. P-values^@^ are calculated for the Total number of inpatients or days.

**Table 2:** Antibiotic prescribing, duration of hospital stay and antibiotic treatment duration within specific diagnosis groups of inpatients at the pediatric departments in a teaching and a non-teaching hospital in Central India.

In each diagnosis group-	Acute Gastroenteritis			Respiratory tract infections			Enteric fever			Viral Fever			Unspecified fever		
	TH	NTH	P	TH	NTH	P	TH	NTH	P	TH	NTH	P	TH	NTH	P
**Total inpatients; n**	168	925		166	1017		41	218		101	185		34	134	
Male; n	106	591		100	743		25	150		60	134		23	87	
Female; n	62	334		66	274		16	68		41	51		11	47	
**Inpatients prescribed AB; n (%)**	51 (30)	695 (75)	*	92 (55)	940 (92)	*	33 (80)	205 (94)	#	32 (32)	130 (70)	*	16 (47)	118 (88)	*
**Duration of hospital stay; mean days (SD)**	5.7 (4.3)	3.6 (1.9)	*	6.6 (4.7)	4.4 (2.1)	*	7.6 (4.3)	4.8 (2.0)	*	5.9 (3.7)	3.9 (1.9)	*	7.3 (6.3)	3.5 (1.5)	*
**Duration of AB treatment; mean days (SD)**	4.8 (2.3)	3.5 (1.8)	*	6.4 (3.7)	4.3 (1.8)	*	6.7 (2.0)	4.8 (2.1)	*	5.3 (2.2)	3.8 (1.6)	*	4.7 (3.3)	3.5 (1.5)	-
**AB prescriptions; n**	356	3077		1039	5023		264	1223		212	532		178	469	
**AB prescription by generic name; n (%)**	226 (64)	151 (5)	*	671 (65)	94 (2)	*	195 (74)	13 (1)	*	147 (69)	15 (3)	*	137 (77)	6 (1)	*
**Number of AB classes prescribed; n**	18	95		27	111		9	62		17	53		11	45	
**DDD/1000 patients**	902	1152		2301	2009		4746	4388		1019	1977		1832	2890	

Abbreviations: TH = teaching hospital; NTH = non-teaching hospital;

P = p values

* = p values ≤0.001

^#^ = p value equal to 0.008

n = number; AB = antibiotics; SD = standard deviation; DDD = defined daily doses.

**Fig 1:**
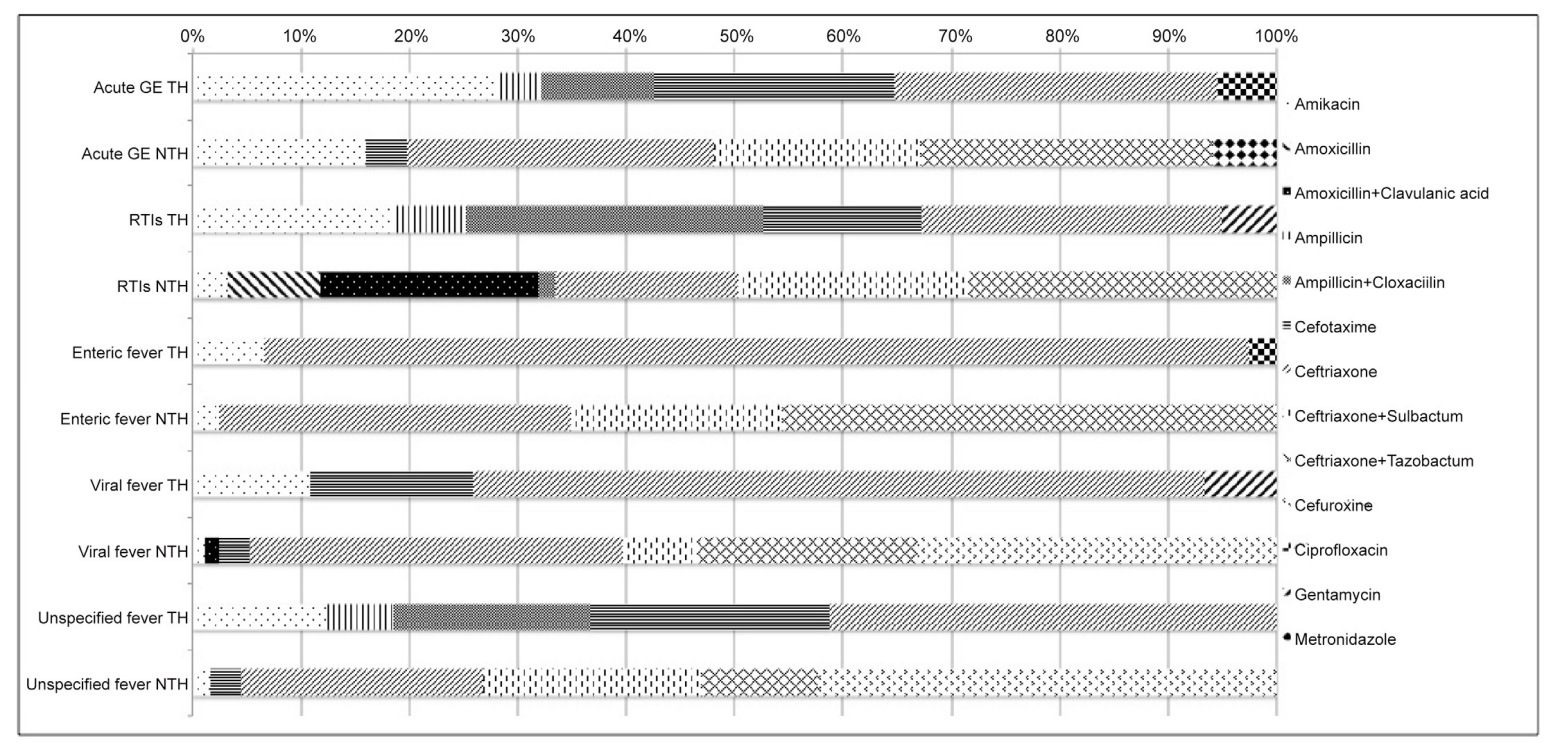
Graphical presentation of defined daily doses of prescribed antibiotics to the inpatients with potential infectious diagnoses at pediatric departments in a teaching- and a non-teaching hospital in India. Abbreviations: DDDs = defined daily doses; GE = gastroenteritis; TH = teaching hospital; NTH = non-teaching hospital; RTIs = respiratory tract infections, DU-drug utilization. 100% in the figure refers to 100% of the DU90 of prescribed antibiotics.

Overall, the parenteral route of administration for antibiotics was dominating prescribed (TH: 96%, 1,976/2,049 prescriptions, NTH: 99%, 10,185/10,324). Both the hospital stay and the duration of antibiotic treatment were significantly longer at the TH for all diagnoses, except for the unspecified fever group (Table 2). Generic name prescribing was more common in the TH for all diagnoses (TH: 67%, 1,376/2,049 versus NTH: 3%, 279/10,324, OR 73.6, 95% CI 63.3 to 85.6; p<0.001)(Table 2). The DU 90% of the prescribed antibiotic classes and substances among the patients with the selected most common diagnoses are presented in Table 3 and Fig 1 respectively. Laboratory diagnostics were rarely used at both study hospitals. Of all antibiotic prescriptions, 37% in the TH versus 24% (p<0.001) in the NTH comprised of antibiotics recommended in the IAP-LEM [8,20].

**Table 3:** DDDs prescribed per thousand inpatients, accounting for 90% of the entire antibiotic prescribing within specific diagnosis groups at the pediatric departments in a teaching and a non-teaching hospital in Central India.

Antibiotic groups and subgroups, ATC	Acute Gastroenteritis		Respiratory tract infections		Enteric fever		Viral Fever		Unspecified fever	
	TH	NTH	TH	NTH	TH	NTH	TH	NTH	TH	NTH
**Antimicrobials for systemic use; J01**	**886**	**1150**	**2301**	**2008**	**4746**	**4388**	**1019**	**1977**	**1832**	**2890**
**β-lactam AB, penicillin; J01C**	**126**	**33**	**747**	**522**	**-**	**225**	**117**	**63**	**447**	**73**
Extended spectrum penicillins	39	21	130	145	-	84	74	36	112	-
FDCs of penicillins incl. β-lactamase AB	87	11	617	376	-	142	43	27	335	73
**Other β- lactam; J01D**	**437**	**402**	**849**	**477**	**4295**	**1536**	**736**	**1358**	**1156**	**1887**
2nd Gen. cephl.	-	-	-	125	-	211	-	605	-	1170
3rd Gen. cephl.	437	400	849	348	4295	1283	736	730	1156	707
**Aminoglycoside; J01G**	**237**	**160**	**463**	**58**	**300**	**94**	**155**	**21**	**228**	**45**
Other Aminoglycosides	237	160	463	58	300	94	155	21	228	45
**Quinolones; J01M**	**46**	**57**	**39**	**79**	**117**	**23**	**8**	**22**	**-**	**-**
Floroquinolones	46	57	39	79	117	23	8	22	-	-
**New fixed dose combinations of AB; J01R**	**-**	**442**	**105**	**861**	**-**	**2465**	**-**	**506**	**-**	**886**

Abbreviations: DDDs = defined daily doses; TH = teaching hospital; NTH = non-teaching hospital; antibacterial = antibiotic; AB = antibiotic; Gen. = generation; cephl = cephalosporins.

### Acute gastroenteritis (AGE)

In the TH, 30% (51/168) patients diagnosed with acute gastroenteritis received antibiotic prescriptions compared to 75% (695/925, p<0.001) in the NTH. The two commonest antibiotics in the TH (constituting >90% of the antibiotic prescribing measured in DDD) were amikacin, (J01GB06: 237 DDD/1000 patients) and ceftriaxone (J01DD04: 250 DDD/1000 patients). In the NTH, ceftriaxone (J01DD04: 266 DDD/1000 patients) was the commonest followed by fixed dose combination (FDC) of ceftriaxone with sulbactum (J01RA84: 178 DDD/1000 patients) and ceftriaxone with tazobactum (J01RA85: 252 DDD/1000 patients, Fig 1). None of the patients in the TH were prescribed a combination of third generation cephalosporin with an extended spectrum β-lactamase (ESBL) inhibitor.

### Respiratory tract infections (RTIs)

In the TH, 55% (92/166) of patients diagnosed with RTIs were prescribed antibiotics, compared to 92% (940/1017, p<0.001) at the NTH. The commonest antibiotics for this diagnosis group were a FDC of ampicillin with cloxacillin (J01CR50: 536 DDD/1000 patients) and ceftriaxone (J01DD04: 540 DDD/1000 patients) in the TH, compared to amoxicillin with clavulanic acid (J01CR02: 340 DDD/1000 patients), ceftriaxone with sulbactum (J01RA84: 360 DDD/1000 patients) and ceftriaxone with tazobactum (J01RA85: 483 DDD/1000 patients) in the NTH (Fig 1 and Table 3). Among the patients with RTIs, 7 (4%) patients in the THand24 (2%) in the NTH were diagnosed with upper respiratory tract infection (URTI). In both hospitals, all patients diagnosed with an URTI were prescribed antibiotics.

### Enteric fever

Among the patients diagnosed with enteric fever, 80% (33/41) in the TH received antibiotic, compared to 94% (205/218, p<0.001) in the NTH. In the TH, ceftriaxone constituted 91% (J01DD04: 4,174 DDD/1000 patients) of the antibiotics prescribed while in the NTH, the commonest antibiotics were ceftriaxone with tazobactum (J01RA85: 1,720 DDD/1000 patients), ceftriaxone (J01DD04: 1,221 DDD/1000 patients) and ceftriaxone with sulbactum (J01RA84: 732 DDD/1000 patients, Fig 1 and Table 3).

### Viral fever

In the TH, 32% (32/101) patients were prescribed antibiotic for diagnosed viral fever compared to 70% (130/185, p<0.001) at the NTH. The commonest antibiotics were ceftriaxone (J01DD04: TH: 601 DDD/1000 patients, NTH: 629 DDD/1000 patients) followed by cefuroxime (J01DC02: 605 DDD/1000 patients) in the NTH. The mean duration of antibiotic treatment among patients with viral infections in the TH was 5.3 days (95% CI- 4.5–6.0 days) and 3.8 days in the NTH (95% CI 3.5–4.0 days).

### Unspecified fever

Among the patients diagnosed with an unspecified fever in the TH, 47% (16/34) were prescribed antibiotics compared to 88% (118/134, p<0.001) at the NTH. The commonest antibiotics were cefotaxime (J01DD01: 403 DDD/1000 patients) and ceftriaxone (J01DD04: 754 DDD/1000 patients) in the TH compared to ceftriaxone (J01DD04: 621 DDD/1000 patients) and cefuroxime (J01DC02: 1,179 DDD/1000 patients) in the NTH (Fig 1 and Table 3).

## Discussion

To our knowledge, this is the first prospective study that describes and compares antibiotic prescribing practices at pediatric departments in Indian private sector hospitals. The study compares the most commonly prescribed antibiotics for a spectrum of potential infectious diagnoses in pediatric departments, namely acute gastroenteritis, respiratory tract infections, enteric fever, viral fever and unspecified fever. This study uses the WHO ATC/DDD methodology. A significantly higher proportion of patients admitted to the pediatric department at the NTH were prescribed antibiotics (84%) compared to the patients at the TH (44%, p<0.001) during the three years study period. Most commonly prescribed groups of antibiotics at the TH were third generation cephalosporins, and new FDCs of antibiotics at the NTH. Both mean duration of stay and mean duration of antibiotic treatment were significantly longer in the TH compared to the NTH.

In absence of any similar published studies from comparable settings globally, we have compared the results of our study with the results of the most comparable settings in this section.

### Antibiotic prescribing and diagnosis groups

Children admitted at the NTH and diagnosed with acute gastroenteritis, viral fever and unspecified fever were prescribed higher DDDs/1000 patients than in the TH. However, a higher proportion of children diagnosed with RTIs and enteric fever were prescribed antibiotics at the TH than in the NTH. Antibiotics are not considered as rational treatment for viral infections and AGE [6,21]. However, a majority of the patients at both hospitals were prescribed antibiotics, both for viral fever and AGE. The patients with viral fever possibly were prescribed antibiotics for superimposed bacterial infection.

### Commonly prescribed classes of antibiotics and factors influencing the prescribing practices

Third generation cephalosporins were the most commonly prescribed antibiotic group at the TH but was the third most prescribed group in the NTH. The patients with enteric fever, AGE and RTIs at both hospitals were commonly prescribed third generation cephalosporins mostly via parenteral route, although it is only recommended for the treatment of complicated or multi drug resistant cases of enteric fever [22]. A previous study conducted among in-patients with infections at the TH also presents that antibiotics were prescribed to the patients of gastroenteritis and respiratory tract infections [14]. According to the WHO, broad-spectrum antibiotics are recommended only when treatment with specific antibiotics has been proven ineffective [1].

New FDCs of antibiotics were more commonly prescribed at the NTH, than at the TH, especially to the patients with AGE, RTIs and enteric fever. Most of the prescribed FDCs at the NTH were irrational combinations. For example a combination of third generation cephalosporins with beta lactamase inhibitors is recommended in infections caused by ESBL producing *E*. *coli* and Klebsiella [14,16]. In addition, the FDCs are usually costlier than single drug formulation thus an extra cost of treatment is directly imposed on the patients [16]. In almost all patients in both study settings, the antibiotics were prescribed empirically but without any laboratory diagnostics, therefore the rationality of the prescribed FDCs could not be evaluated. The probable unnecessary exposure to antibiotics for viral infections and broad spectrum antibiotics including FDCs could potentially lead to the development of antibiotic resistance. Factors influencing the antibiotics prescribing practices are multi-factorial and will be addressed below.

#### a. Parents' pressure

It is known that physicians perceiving prescribing pressure from the parents prescribe antibiotics more often [23,24]. An Indian study from Delhi showed that doctors' perceived that demand and expectations from the parents contributed to the prescribing of antibiotics [25]. The doctors expressed that patients who were charged a fee for medical consultation and services had high expectations for receiving antibiotic prescriptions The pressure was felt to prescribe antibiotics to the patients presenting with fever or gastroenteritis rather than embrace a wait-and-see policy due to the fear of losing the patient for future consultancies [25]. In our study, the parents at the NTH had paid for the treatment, thus perceived pressure from the parents might be considered as a contributory factor for higher antibiotic prescribing rates at the NTH than in the TH where the medical services were free of charge.

#### b. Pressure from pharmaceutical companies and prescribers' profit motive

Generic name prescribing is preferred over trade names for easy availability and cost effectiveness. The generic medicines, compared to the brand medicines, also provide flexibility to choose and dispense from the available formulations and promote access to the world’s poorest population. According to the WHO, rational prescribing of medicines includes prescribing by generic names rather than by the trade names [12]. In the present study, trade names were used at both hospitals however; almost all prescriptions at the NTH were by trade names.

A study conducted in Bangkok, Thailand included three public hospitals (one teaching hospital) and three private not for-profit non-teaching hospitals. The public teaching hospital and private not for-profit hospitals could be broadly compared with our TH and NTH respectively. The use of trade names in the NTH was substantially higher than the private not for-profit hospitals (82%) in the Thai study [26]. The prescribing patterns at the Thai non-teaching hospital were considered as an effect of prescribers’ profits as well as their contact with pharmaceutical marketing representatives [26]. Similarly high antibiotic prescribing rates, excessive prescribing of broad-spectrum and newer FDCs of antibiotics and use of trade names at the NTH might be related to potential pressure from pharmaceutical companies. This is further supported by a lower trade name prescribing at the TH as medical representatives' visits are restricted due to hospital policy and a hospital based study conducted for 5 months in the region [15].

The influence of pharmaceutical companies over high antibiotic prescribing has been reported in studies conducted worldwide [15,25–27]. A study among prescribers in the United States shows that the prescribers who had been exposed to the medical representatives, favoured trade name prescribing, rather than generic name prescribing or nondrug treatment options with comparable effects [27].

Similarly, a study of antibiotic prescribing practices in Delhi showed a tendency to prescribe newer antibiotics at private sector clinics and its association with a profit-motive for the prescribers [13].

#### c. Educational activities

The influence of educational profile and ongoing academic activities at the TH might have resulted in higher generic name prescribing since teaching hospitals are associated with educational institutions and are expected to be more updated about the prescribing guidelines and recommendations through continuing medical education (CME) programs, regular schedules of seminars, teaching and inter- and intra- departmental meetings [16].

#### d. Adherence to the available guidelines

The results show that prescribing of essential medicines from the IAP-LEM was higher at the TH [8]. At the TH, 37% of the antibiotic prescriptions comprised of antibiotics recommended in the IAP-LEM, compared to 24% at the NTH. Earlier studies have shown a reduction of broad-spectrum antibiotic use in health care facilities after implementation of local prescribing guidelines [9,28]. Since the long-term aim of the study is to develop local prescribing guidelines and to implement them at the pediatric departments at both hospitals, a reduction in irrational antibiotic prescribing at the study settings might be expected in the future.

In the Thai study, a higher proportion of antibiotics were present from the list of essential antibiotics, at the teaching hospital compared to the non-teaching hospitals (56% vs. 43%) [26].

#### e. Antibiotic treatment duration and route of administration

The hospital stay and antibiotic treatment duration was significantly longer for pediatric inpatients at the TH than at the NTH. The Thai study concluded that the patients in public teaching hospital had longer antibiotic treatment durations compared to the patients in private hospitals [26]. The study also showed longer treatment durations at a teaching hospital (7.2 days) compared to the non-teaching hospitals (4.2 days) [26]. These results are comparable to our study, where antibiotic treatment duration was significantly longer in pediatric inpatients at the TH than at the NTH. An antibiotic treatment duration of 5 to 7 days is recommended for children with mild or moderate forms of community acquired pneumonia or up to 14 days for severe pneumonia [29, 30].

In a study conducted among pediatric patients in Uttar Pradesh, India, parenteral route administration of antibiotics was higher in private health care facilities compared to the public facilities [5]. This result was in concordance to the results of our study, where almost all antibiotics were administered parenterally at both hospitals. The reason behind this practice could be explained as the patients in the department are mainly young and sick thus might have difficulty in oral administration of medicines.

#### f. Use of laboratory services

Earlier studies of antibiotic prescribing for pediatric patients in Indian healthcare facilities show that prescribers tend to consider fever as a sign of a bacterial infection and prescribe antibiotics [5,14]. In the present study, the doctors neither at the TH nor at the NTH routinely sent samples for microbiology testing, which means that they might also have prescribed antibiotics symptomatically and continued with the empiric treatment without any evidence based adjustments. Antibiotics are rational treatment when a correct substance is prescribed for an appropriate indication with appropriate dose, frequency and duration. Thus, as no laboratory confirmatory diagnostics were performed to adjust the initial empiric therapy it is possible that some pediatric patients in both hospitals might have been prescribed antibiotics unnecessarily or that the wrong antibiotic was used.

Inappropriate antibiotic prescribing can be reduced by better use of laboratory based diagnostics or point of care (POC) diagnostics. However, cost effective POC diagnostics that differentiate bacterial and viral infections are not easily available. In both the settings of our study, laboratories were underutilized both for biochemical and microbiological confirmation of bacterial infections, despite of easy and free availability. The reasons for underutilization of the laboratory facilities need to be explored further.

### Methodological considerations

Since antibiotic prescribing data were prospectively recorded from all inpatients at the pediatric departments, the study populations were representative for the pediatric patients admitted to the hospitals. Data were comparable between the hospitals since the same form for data collection was used at both hospitals. At both hospitals, the nurses were trained for data collection to ensure the quality of data. The same person supervised the training and data collection process in both hospitals. Despite this, in view of high turnover of nurses and because data collection was done manually the risk of missing data of some patients could not be denied. This was an observational study with no intention to intervene in the on-going treatment, thus the final diagnoses registered for each patient were not confirmed by results from microbiology tests, unless ordered by the consultant in-charge. Further, the aim of the study was to present the pattern of antibiotic prescribing thus, the confirmation of diagnosis was beyond the aim. As a result some patients might have been incorrectly diagnosed. The absence of results from microbiology tests makes it difficult to decide whether antibiotic treatment was rational or not. Measurement of the amount of antibiotic prescribing among pediatric patients is a challenge since the DDD are based on the antibiotic dose in adults, but as it is a technical unit intended for comparisons it can still be used for the comparative purposes as the ages of patients were similar in both study hospitals. Also DDD is internationally considered to be the best method for comparisons of antibiotic prescribing [16,23].

## Conclusions

Overalls significant differences in prescribing patterns of antibiotics between the TH and the NTH were seen in terms of percentage of patients prescribed antibiotics, duration of stay and antibiotic treatment and use of generic name in the prescriptions, for the 5 most common diagnoses studied in present communication. The antibiotic prescribing was high for all inpatients at both hospitals, but a lower proportion of patients were prescribed antibiotics at the TH compared to the NTH. Broad-spectrum antibiotics were prescribed frequently at both hospitals but third generation cephalosporins were most commonly prescribed at the TH and new FDCs of antibiotics at the NTH. There was a significant difference in the proportion of patients with RTIs prescribed antibiotics between the two hospitals. Compliance to the IAP-LEM for children and generic name prescribing was higher at the TH than NTH.

This study highlights need for development and implementation of relevant, diagnosis-specific antibiotic prescribing guidelines for pediatric inpatients. The development of a pediatric DDD is suggested which would enable appropriate comparisons of antibiotic prescribing among the pediatric inpatients.

There is need for development of evidence based treatment protocols for common clinical conditions to rationalize the use of antibiotics, as indicated in this paper.

## Abbreviations

AGE: Acute gastroenteritis
ATC: Anatomical therapeutic chemical
CME: Continuing medical education
DDD: Defined daily dose
DU: Drug utilisation
ESBL: Extended spectrum β-lactamase
FDCs: Fixed dose combinations of antibiotics
IAP-LEM: Indian academy of pediatrics list of essential medicines for children in India
NICU: Neonatal intensive care unit
NTH: Non-teaching hospital
POC: Point of care
RTI: Respiratory tract infection
TH: Teaching hospital
URTI: Upper respiratory tract infection
WHO: The World Health Organisation
WHOCC: The World Health Organisation Collaborating Centre for Drug Statistics Methodology
